# Cutaneous Silicone Granuloma Mimicking Breast Cancer after Ruptured Breast Implant

**DOI:** 10.1155/2011/129138

**Published:** 2012-01-26

**Authors:** Waseem Asim Ghulam El-Charnoubi, Trine Foged Henriksen, Jens Joergen Elberg

**Affiliations:** Department of Plastic Surgery, Breast Surgery and Burn Care, Rigshospitalet, Blegdamsvej 9, 2100 copenhagen, Denmark

## Abstract

Cutaneous manifestations due to migration of silicone from ruptured implants are rare. Migrated silicone with cutaneous involvement has been found in the chest wall, abdominal wall, and lower extremities. We describe a case of cutaneous silicone granuloma in the breast exhibiting unusual growth mimicking breast cancer after a ruptured implant.

## 1. Introduction

Since the introduction of silicone implants in 1963, the implants have undergone considerable product development increasing their safety. However, at least 15% of former generations of breast implants have been found to rupture before the 10th year after implantation [1]. It is well known that extracapsular rupture increases the risk for silicone migration to distant sites and thus formation of peripheral silicone granulomas [2].

Highly cohesive silicone implants were introduced in 1993, and it is expected that the silicone in these types of implants does not leak into the surrounding tissue and does not disperse to distant sites [3]. Distant migration of silicone in these types of implants has not been described in the literature.

## 2. Case Report

**Figure 1:**
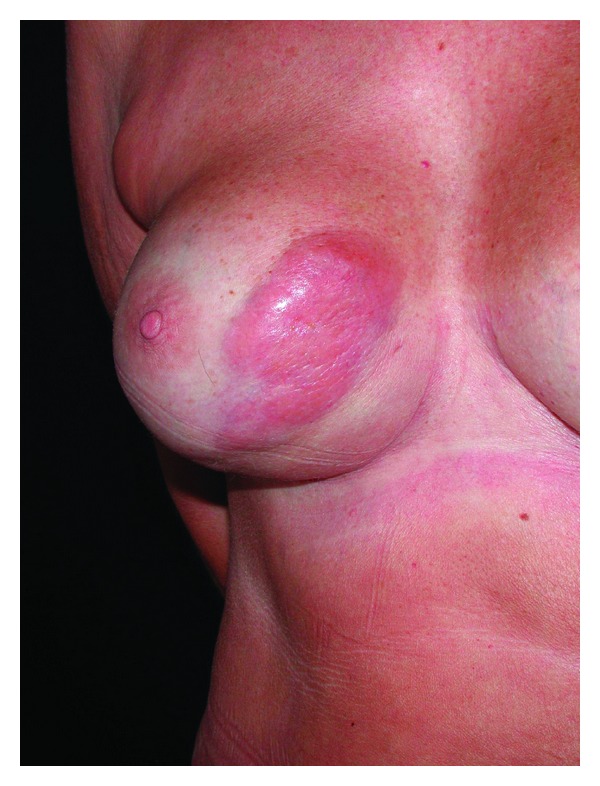
Preoperative view of patient with swelling and redness of the right medial part of the breast.

A 60-year-old woman was referred to our outpatient clinic with a tumor in the right lower medial quadrant of the breast. The symptoms began 7-8 years earlier, now presenting with redness and swelling of the overlying skin (Figure 1).

**Figure 2:**
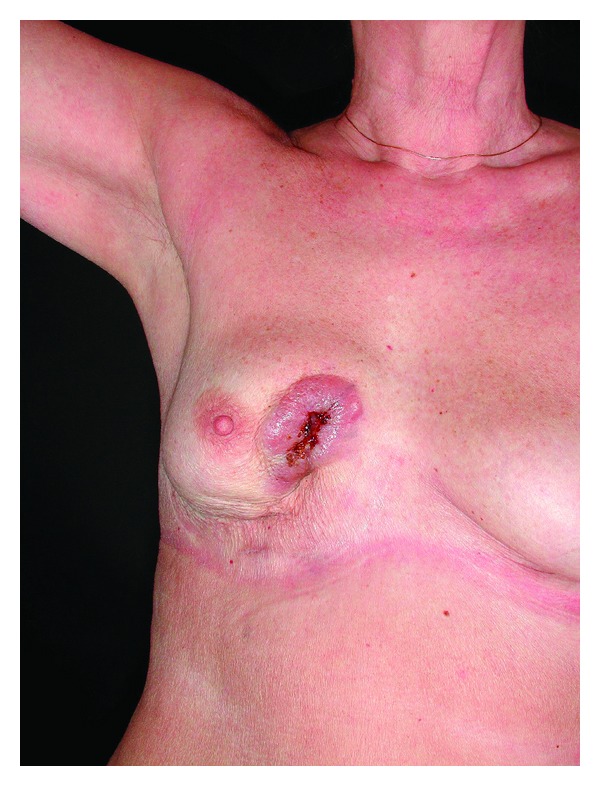
3 months after explantation with ulceration.

The patient had undergone breast augmentation, with silicone implants 13 years earlier. The patient was initially referred because of suspected breast cancer. Biopsies confirmed silicone granuloma. Ultrasound confirmed bilateral rupture and possible extracapsular rupture on the right side. MRI confirmed bilateral intracapsular rupture with “nose sign and linguine sign” and extracapsular rupture on the right side with extensive leakage of silicone involving the skin. During surgery, silicone granuloma was found from the periprosthetic capsule into the overlying skin and multiple cavities containing silicone. The implants were removed, and thoroughly capsulectomy was performed. After 3 months, the patient presented a tender and ulcerated tumor in the right lower medial quadrant (Figure 2).

**Figure 3:**
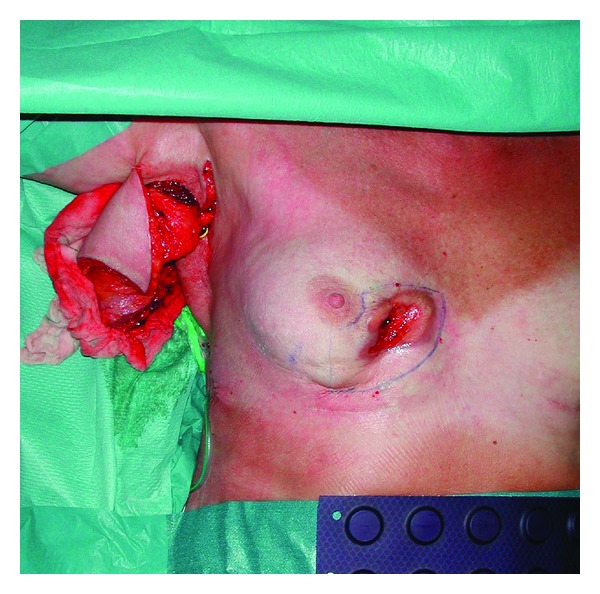
Area of excision is marked, and the flap has been raised.

**Figure 4:**
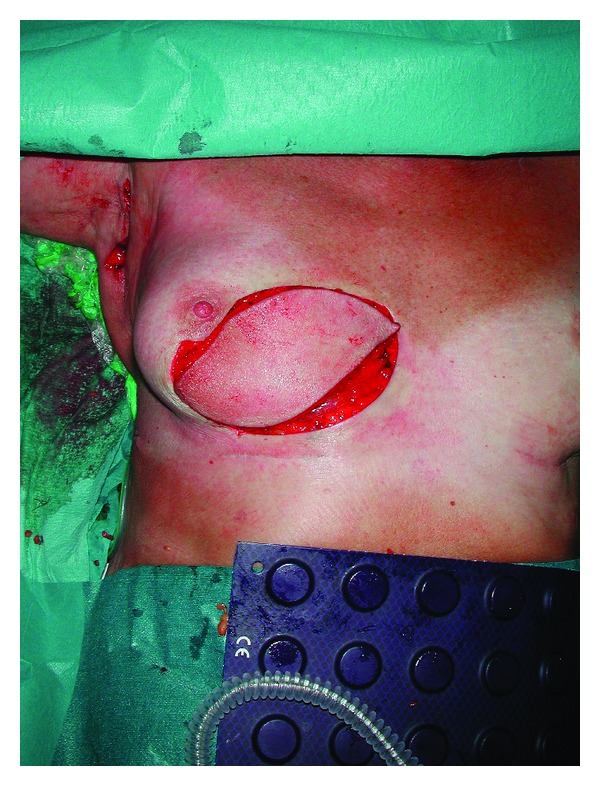
The flap has been placed in the defect together with the breast implant.

**Figure 5:**
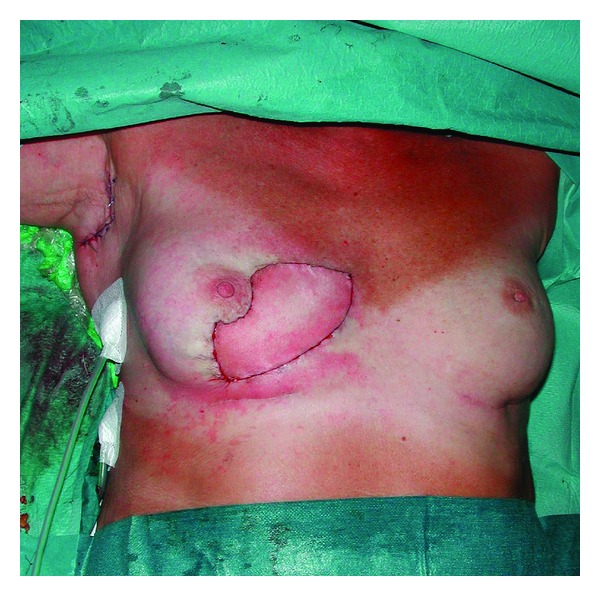
The final result.

A biopsy was again performed because of suspected malignancy, and once again pathological examination showed silicone granuloma. The patient underwent additional surgery, and the silicone granuloma was excised including a major part of the overlying skin. The breast was reconstructed with a latissimus dorsi musculocutaneous flap and a highly cohesive implant (Figures 3, 4 and 5).

## 3. Discussion

Silicone granuloma (SG) or siliconoma was first described in 1964 by Winer et al. after injection of free silicone for breast augmentation and as facial filler [4].

SG in relation to ruptured silicone implants, were first described in the 1980s [5]. SG can occur in all sizes from microscopic to the clinically recognizable and symptomatic. It is a foreign body reaction often found around the area of the capsule surrounding the implant [6].

Austad describes SG as a rare phenomenon, not always caused by dehisced implants [7]. In surveys of explanted women, extracapsular leakage is mentioned in 3.2%, without SG being noticed [8]. It is unclear why some patients develop SG, while others despite dehisced implants do not develop these. There are several theories on SG formation, including infection with low-pathogenic bacteria and “in vivo” modification of silicone structure over time [7, 9]. Studies have shown that alteration of the polymer structure results in a less viscous silicone, enabling the distant migration [9]. It has been suggested that silicone migrate through lymphatic or blood vessels [10]. To prevent further migration, resection is recommended. Case reports describe patients with SG where hesitancy led to continuous growth and perforation of SG to the skin, chronic pain, neuropathy and scarring [11, 12]. It is not clear why some SG behaves more aggressively.

In our case, the SG behaved like a neoplastic process, continuing growing even after the explantation suggesting that a granulomatous inflammatory reaction continued in the overlying skin.

Usually implant failure is found in the “older generations” of silicone implants, so this phenomenon will probably diminish in the future due to the use of highly cohesive implants. Dragu et al. suggest replacing all “old” implants with the modern highly cohesive silicone implants [13].
